# Skeletal muscle TET3 promotes insulin resistance through destabilisation of PGC-1α

**DOI:** 10.1007/s00125-023-06073-5

**Published:** 2024-01-13

**Authors:** Beibei Liu, Di Xie, Xinmei Huang, Sungho Jin, Yangyang Dai, Xiaoli Sun, Da Li, Anton M. Bennett, Sabrina Diano, Yingqun Huang

**Affiliations:** 1 Department of Obstetrics, Gynecology & Reproductive Sciences, Yale University School of Medicine, New Haven, CT USA; 2 Center of Reproductive Medicine, National Health Commission Key Laboratory of Advanced Reproductive Medicine and Fertility, Shengjing Hospital of China Medical University, Shenyang, China; 3 Present Address: Department of Reproductive Medicine, General Hospital of Central Theater Command, Wuhan, Hubei China; 4 Present Address: Department of Endocrinology, Fifth People’s Hospital of Shanghai, Fudan University School of Medicine, Shanghai, China; 5 Institute of Human Nutrition, Columbia University Irving Medical Center, New York, NY USA; 6 Assisted Reproduction Unit, Department of Obstetrics and Gynecology, Sir Run Run Shaw Hospital, School of Medicine, Zhejiang University, Hangzhou, China; 7 Present Address: Center of Reproductive Medicine, Department of Obstetrics and Gynecology, Affiliated Hospital of Nantong University, Jiangsu, China; 8 Departments of Pharmacology and of Comparative Medicine, Yale University School of Medicine, New Haven, CT USA; 9 Yale Center for Molecular and Systems Metabolism, Yale University School of Medicine, New Haven, CT USA

**Keywords:** Diabetes, Insulin resistance, Mitochondria, Obesity, PGC-1α, Skeletal muscle, TET3

## Abstract

**Aim/hypothesis:**

The peroxisome proliferator-activated receptor-γ coactivator α (PGC-1α) plays a critical role in the maintenance of glucose, lipid and energy homeostasis by orchestrating metabolic programs in multiple tissues in response to environmental cues. In skeletal muscles, PGC-1α dysregulation has been associated with insulin resistance and type 2 diabetes but the underlying mechanisms have remained elusive. This research aims to understand the role of TET3, a member of the ten-eleven translocation (TET) family dioxygenases, in PGC-1α dysregulation in skeletal muscles in obesity and diabetes.

**Methods:**

TET expression levels in skeletal muscles were analysed in humans with or without type 2 diabetes, as well as in mouse models of high-fat diet (HFD)-induced or genetically induced (*ob*/*ob*) obesity/diabetes. Muscle-specific *Tet3* knockout (mKD) mice were generated to study TET3’s role in muscle insulin sensitivity. Genome-wide expression profiling (RNA-seq) of muscle tissues from wild-type (WT) and mKD mice was performed to mine deeper insights into TET3-mediated regulation of muscle insulin sensitivity. The correlation between PGC-1α and TET3 expression levels was investigated using muscle tissues and in vitro-derived myotubes. PGC-1α phosphorylation and degradation were analysed using in vitro assays.

**Results:**

TET3 expression was elevated in skeletal muscles of humans with type 2 diabetes and in HFD-fed and *ob*/*ob* mice compared with healthy controls. mKD mice exhibited enhanced glucose tolerance, insulin sensitivity and resilience to HFD-induced insulin resistance. Pathway analysis of RNA-seq identified ‘Mitochondrial Function’ and ‘PPARα Pathway’ to be among the top biological processes regulated by TET3. We observed higher PGC-1α levels (~25%) in muscles of mKD mice vs WT mice, and lower PGC-1α protein levels (~25–60%) in HFD-fed or *ob*/*ob* mice compared with their control counterparts. In human and murine myotubes, increased PGC-1α levels following TET3 knockdown contributed to improved mitochondrial respiration and insulin sensitivity. TET3 formed a complex with PGC-1α and interfered with its phosphorylation, leading to its destabilisation.

**Conclusions/interpretation:**

Our results demonstrate an essential role for TET3 in the regulation of skeletal muscle insulin sensitivity and suggest that TET3 may be used as a potential therapeutic target for the metabolic syndrome.

**Data availability:**

Sequences are available from the Gene Expression Omnibus (https://www.ncbi.nlm.nih.gov/geo/) with accession number of GSE224042.

**Graphical Abstract:**

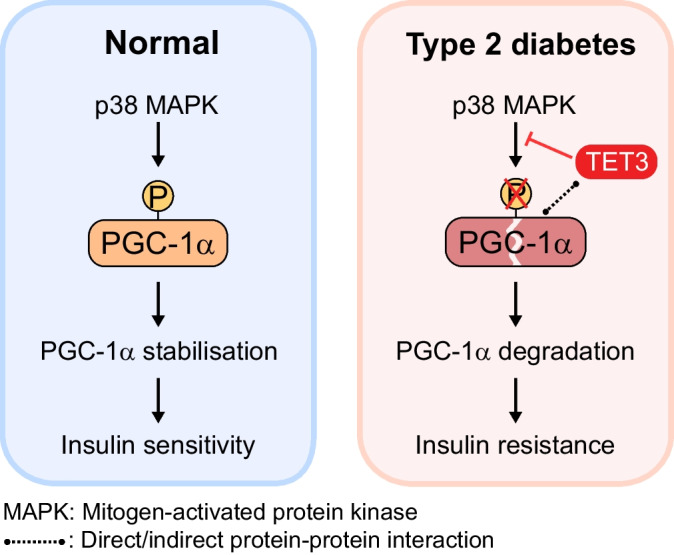

**Supplementary Information:**

The online version contains peer-reviewed but unedited supplementary material available at 10.1007/s00125-023-06073-5.


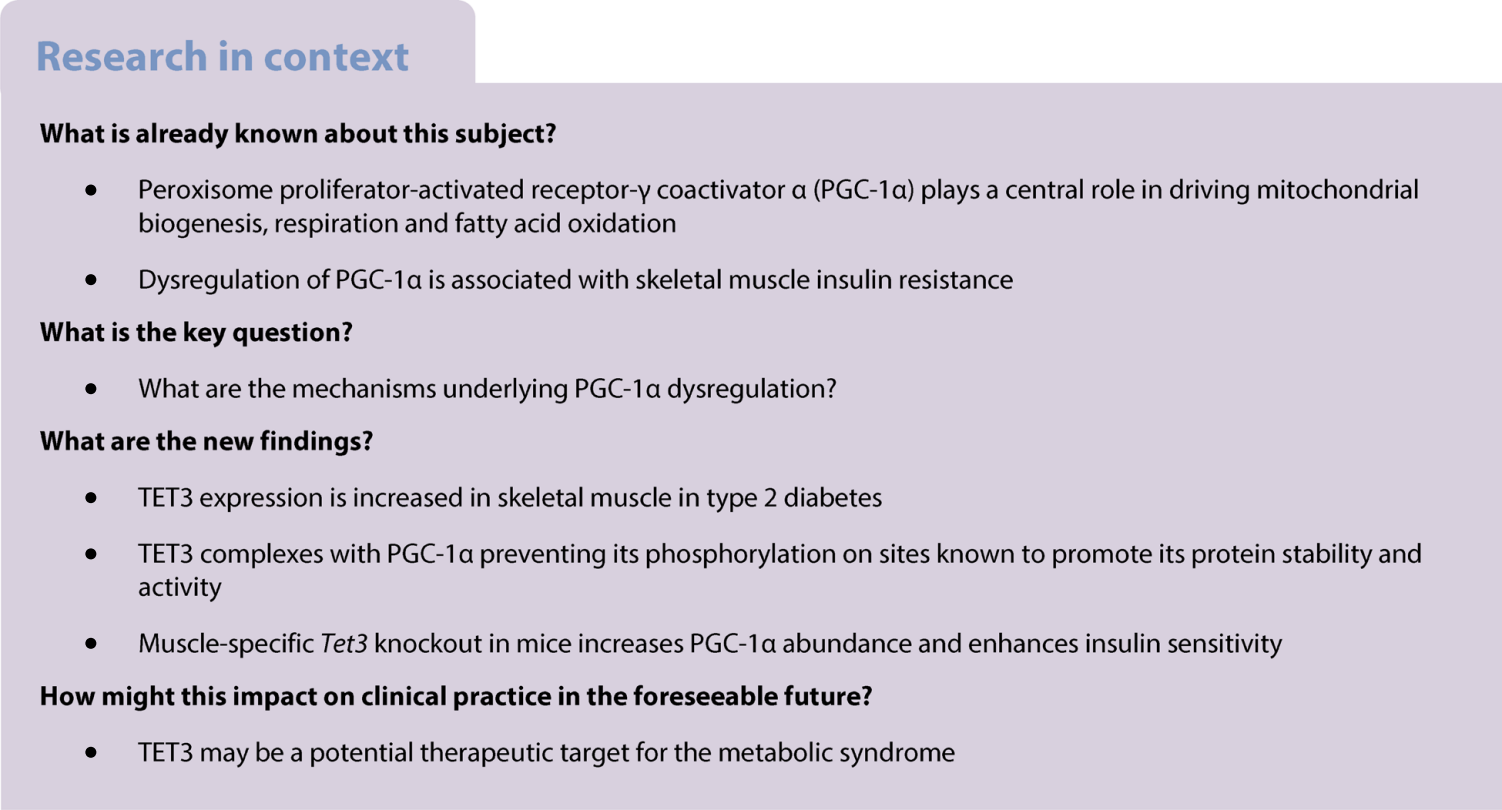


## Introduction

The hallmark of type 2 diabetes is persistent hyperglycaemia resulting from a combination of peripheral insulin resistance and inappropriate secretion of insulin and glucagon from the pancreas [1]. Chronic hyperglycaemia in combination with other metabolic aberrations causes damage to organs and blood vessels and substantially increases the risk for various complications, leading to premature disability and death [1, 2]. Skeletal muscle is the major tissue responsible for insulin-stimulated glucose disposal and muscle insulin resistance is a critical component in the pathogenesis of type 2 diabetes [3–5]. While the exact causes of muscle insulin resistance remain incompletely understood, available literature points to dysregulation of peroxisome proliferator-activated receptor-γ coactivator α (PGC-1α) as an important mechanism. As a transcriptional coactivator, PGC-1α plays a critical role in the maintenance of glucose, lipid and energy homeostasis by orchestrating metabolic programs in response to numerous environmental stimuli (e.g. nutrients, temperature, exercise) in a tissue-specific manner [6, 7]. Highly expressed in skeletal muscle, PGC-1α has been implicated in the regulation of energy metabolism, myofibre composition, skeletal muscle mass, angiogenesis and neuromuscular junction remodelling [7–9]. The best studied role of PGC-1α in myocytes is its ability to powerfully drive the transcriptional program of mitochondrial biogenesis, respiration and fatty acid β-oxidation through co-ordinately activating a wide variety of transcription factors including nuclear respiratory factors, oestrogen related receptors, and peroxisome proliferator-activated receptor (PPAR) family members [7, 8, 10]. An additional role of PGC-1α is to promote fatty acid uptake and lipid biosynthesis by upregulating a set of genes involved in these processes. Given the implication of myocyte overload of lipid/intermediate lipid metabolites in causing insulin resistance, a fine balance between the two actions of PGC-1α (fatty acid uptake/lipid biosynthesis vs fatty acid oxidation) is critical to metabolic health. Indeed, supraphysiological muscle overexpression of PGC-1α paradoxically induces intramuscular lipid accumulation and insulin resistance in transgenic mice [11, 12], whereas modest increases (~25%) in PGC-1α protein abundance in muscle promote insulin sensitisation [9, 13–15]. Importantly, repression of the PGC-1α-dependent mitochondrial program has long been documented in skeletal muscles of individuals with type 2 diabetes, and PGC-1α dysregulation and hence mitochondrial insufficiency are widely acknowledged contributors to muscle insulin resistance [9, 16–19]. However, the causes of PGC-1α dysregulation have remained elusive.

The ten-eleven translocation (TET) family of dioxygenases (TET1, TET2 and TET3) regulate gene expression by oxidising methylated cytosine (5mC) to 5-hydroxymethylcytosine (5hmC) and further oxidised derivatives, leading to DNA demethylation [20, 21]. TET-induced epigenetic regulation can also occur in an enzymatic activity-independent manner [22–28]. Despite the importance of TETs in development, cancer, stem cells and immunity [20, 24, 25, 29–33], their roles in energy metabolism have just begun to be recognised, as exemplified by a few recent publications [27, 28, 34–36]. We were the first to report that TET3 expression is increased in the livers of humans with type 2 diabetes and in mouse models of type 2 diabetes, contributing to hyperglycaemia [34]. Mechanistically, TET3 induces promoter demethylation of the transcription factor, hepatocyte nuclear factor 4α (HNF4α), promoting hepatic glucose production [34]. We later reported that the hepatic TET3/HNF4A regulatory pathway underlies the therapeutic effects of metformin [35]. Recently, a role for TETs in adipose tissues in energy expenditure has also been uncovered. In mouse adipocytes, it was shown that TET1 coordinates with histone deacetylase 1 (HDAC1) to epigenetically suppress thermogenic gene transcription and that adipocyte-specific *Tet1* knockout in mice increases energy expenditure and protects against diet-induced obesity and insulin resistance [27]. Likewise, adipose-specific ablation of all three *Tet* genes enhances β-adrenergic responses, increases energy expenditure and protects against obesity [28]. Mechanistically, TETs suppress the transcription of β_3_-adrenergic receptor by recruiting histone deacetylases to its promoter [28]. Further, we have recently documented that CRISPR-mediated, agouti-related protein (AgRP) neuron-specific *Tet3* ablation induces hyperphagia, systemic insulin resistance, obesity and type 2 diabetes [36]. Mechanistically, we reported a dynamic association of TET3 with the *Agrp* promoter in response to leptin signalling that induces 5hmC modification and association of a chromatin-modifying complex, leading to transcription inhibition of *Agrp*. Importantly, this regulation occurs both in mouse models and human cells [36]. In the current work we report that TET3 regulates skeletal muscle insulin sensitivity through a novel mechanism of action.

## Methods

### Animals

All animal work was approved by the Yale University Institutional Animal Care and Use Committee. All mice used in this report were female. Mice were housed at 22–24°C under a 12 h light–dark cycle and were fed with regular chow (RC) (Harlan Teklad no. 2018, IN, USA; 18% energy from fat) or high-fat diet (HFD) (Research Diets, NJ, USA, D12451; 45% energy from fat); water was provided ad libitum. C57BL/6J (Jax, CT, USA, 000664), HAS-Cre79 (Jax, 006149) and *Lep*^ob/ob^ (Jax, 000632) mice were purchased from the Jackson Laboratory. The *Tet3*^fl/fl^ mice were generous gifts from A. Rao from La Jolla Institute for Immunology (CA, USA). The muscle-specific *Tet3* knockout (mKD) mice were created by crossing *Tet3*^fl/fl^ mice with mice expressing Cre recombinase under the control of human α skeletal muscle actin (HSA) promoter (HSA-Cre79 mice). TET3 floxed littermates (wild-type [WT]) were used as controls. Randomisation was not feasible during group assignment, but results were analysed in a blinded manner whenever possible. Data were not included if values were excluded by outlier test. For information on animal numbers, refer to figure legends. Before experiments, mice were allowed to acclimate for at least 7 days in our animal facility.

### Body composition and indirect calorimetry analyses

Body composition was assessed by MRI (EcoMRI; Echo Medical Systems, TX, USA). Food intake, energy expenditure and locomotor activity were measured using an indirect calorimetry chamber (TSE Systems, Germany).

### GTT and ITT

The GTT and ITT were conducted as previously described [35]. For the GTT, following a 14 h overnight fast, each mouse received an i.p. injection of 2 g/kg body weight glucose (Sigma-Aldrich, MO, USA, G5767) in sterile saline (154 mmol/l NaCl). Blood glucose concentrations were measured using Contour next blood glucose meter (Ascensia Diabetes Care, NJ, USA) via tail vein bleeding at the indicated time points after injection. The ITT was performed in ad libitum fed mice. Each mouse received an i.p. injection of insulin, 1 U/kg (Humulin R; Eli Lilly, MA, USA). Blood glucose concentrations were measured using Contour next blood glucose via tail vein bleeding at the indicated time points after injection.

### Hyperinsulinaemic–euglycaemic clamp studies

The hyperinsulinaemic–euglycaemic clamp studies were performed using previously described methods [34]. Briefly, mice at the age of 12 weeks were catheterised in jugular veins with polyethylene catheters under deep anaesthesia. Mice were singly housed for a 4 day recovery period after surgery. The clamp experiments were performed using conscious and unrestrained mice after 16 h overnight fasting. The protocol consisted of a 120 min basal period (*t*=−120–0 min) followed by a 115 min clamp period (*t*=0–115 min). [3-^3^H]Glucose (185,000 Bq; Perkin Elmer, MA, USA) was given at *t*=−120 min followed by a 1850 Bq/min infusion for 2 h. During the basal period, at *t*=−15 min and *t*=−5 min, blood samples were taken for the assessment of basal glucose level and glucose turnover. The clamp period was begun at *t*=0 min with primed and continuous infusion of human insulin (8 mU/kg bolus followed by a rate of 2.5 mU kg^–1^ min^–1^; Humulin R; Eli Lilly). Blood glucose was measured by glucometer (Breeze 2; Bayer HealthCare, NJ, USA) at 10 min intervals, and 30% glucose was infused at a variable rate in order to maintain euglycaemic levels (6.1–7.2 mmol/l). Blood samples were collected every 10 min from *t*=70 min to *t*=115 min and processed to determine glucose specific activity. Following collection of the final blood sample, the mice were euthanised and tissues were harvested, frozen in liquid nitrogen and stored at −80°C until later use.

### Myoblast culture and differentiation

Undifferentiated mouse C2C12 myoblasts (Sigma-Aldrich, 91031101-iVL) were maintained in growth medium (DMEM [Gibco, NY, USA, 11965-092] supplemented with 10% (vol./vol.) FBS, heat inactivated, 1% (vol./vol.) penicillin/streptomycin, 1% (vol./vol.) l-glutamine and 1 mmol/l sodium pyruvate). Cells were authenticated and free of mycoplasma. To prepare for differentiation, cells were seeded at a density of 2.0×10^4^ cells/well in growth medium in 24-well plates. Differentiation was initiated 2 days later when cells became confluent by replacing growth medium with differentiation medium containing 2% horse serum (16050-130) in place of 10% FBS. The medium was changed every other day until transfection, which was performed on day 4 or 5 after initiation of differentiation. Cryopreserved primary human skeletal myoblasts (HSkMs) (Thermo Fisher Scientific, MA, USA, A11440) were purchased. To prepare for differentiation, cells were thawed in Gibco HSkM Differentiation Medium (DMEM Basal Medium [11885-084] supplemented with 2% horse serum) and seeded in a 24-well plate at a density of 2.4×10^5^ cells/well in the differentiation medium. Cells were incubated in a tissue culture incubator for 48 h to allow rapid differentiation, followed by siRNA transfection.

### siRNA transfection

To prepare siRNA transfection solution for each well of cells in a 24-well plate, 20 pmol of non-targeting control siRNA (NT siRNA) (Ambion, CT, USA, AM4636), siRNA specific for mouse *Tet3* (*Tet3* siRNA; Ambion, 4390815/s101483), mouse *Pgc1a* (*Pgc1a* siRNA; Ambion, 4390771/n253420), human *TET3* (*TET3* siRNA; Ambion, 4392420/s47239) or human *PGC1A* (*PGC1A* siRNA; Ambion, 4392420/s21394) was mixed with 100 μl of OPTI-MEM (Gibco, 31985-070) by gentle pipetting. In parallel, 6 μl of Lipofectamine RNAiMAX (Invitrogen, MA, USA, 13778-150) was mixed with 100 μl of OPTI-MEM by gentle pipetting, then the two were combined. Following 5 min of incubation at room temperature, the resulting 200 μl of transfection solution was added to each well of cells. For treatment with NT siRNA alone, 20 pmol of NT siRNA was used for each well of cells. For treatment with TET3 siRNA alone, 10 pmol of NT siRNA and 10 pmol of *TET3* siRNA (or *Tet3* siRNA) were used for each well of cells. For TET3/PGC-1α double knockdown, 10 pmol of *TET3* siRNA (or *Tet3* siRNA) and 10 pmol of *PGC1A* siRNA (or *Pgc1a* siRNA) were used for each well of cells. Therefore, the total amount of siRNAs for each well of cells was 20 pmol. After 12 h of incubation at 37°C in a 5% humidified CO_2_ tissue culture incubator, 300 μl of medium was added and incubation was continued for an additional 24 or 48 h until further analyses.

### PGC-1α protein stability assay

C2C12 myoblasts were transfected with NT siRNA or *Tet3* siRNA as described above for 48 h, followed by addition of cycloheximide (CHX) (Cell Signaling Technology, MA, USA 2112) at a final concentration of 50 μg/ml. Proteins were isolated at 0, 15, 30 and 45 min later and analysed by western blotting using anti-PGC-1α (dilution 1:1000; Proteintech, IL, USA, 66369-1-Ig) and horseradish peroxide (HRP)-conjugated anti-GAPDH (dilution 1:5000; Proteintech, HRP-60004).

### Western blot analysis

To extract proteins from cultured cells, myoblasts or myotubes were homogenised in situ using a pipette tip in 2 × SDS sample buffer with 10% β-mercaptoethanol at room temperature in less than 5 s followed by heating at 100°C for 5 min with occasional vortex. To extract proteins from muscle tissues, frozen tissue samples (~50 mg) were homogenised in 200 μl of tissue lysis buffer (15% SDS, 75 mmol/l Tris HCl, pH 7.4, 1× protease inhibitor cocktail [Thermo, 78438], 1× phosphatase inhibitor cocktail [Thermo, 78427], 5% β-mercaptoethanol) using a BeadBug6 Microtube homogeniser (Benchmark) set at speed 3600, 20 s on and 20 s off for six cycles. The lysate was cooled down on ice for 10 min, followed by centrifugation at 12,000 *g* at 4°C for 7 min to remove insoluble materials. The supernatant fraction was transferred to a new tube and glycerol (final concentration 20%) and bromophenol (for tracking purpose during gel running) were added. Samples were then heated at 100°C for 5 min with occasional vortex, aliquoted, and stored at −80°C until use. Tissue samples were freshly diluted at 1:1 – 1:3 in 2 × SDS sample buffer before loading. Cell and tissue samples were loaded at 5–10 μl per well onto 4–15% gradient SDS gels (Bio-Rad), followed by western blot analysis. The antibodies used were anti-TET3 (for mouse TET3; dilution 1:1000; Active motif, CA, USA, 61395), anti-TET3 (for human TET3; dilution 1:1000; GeneTex, CA, USA, GTX121453), anti-PGC-1α (dilution 1:1000; Proteintech, 66369-1-Ig), anti-phospho-Ser265 (dilution 1:1000), anti-phospho-Thr298 (dilution 1:1000), anti-TET2 (dilution 1:1000, Proteintech, 21207-1-AP) and HRP-conjugated anti-GAPDH (dilution 1:5000; Proteintech, HRP-60004). The secondary antibodies were HRP-linked anti-rabbit IgG (dilution 1:10,000; Rockland, PA, USA, 611-1322) and HRP-linked anti-mouse IgG (dilution 1:10,000; Cell Signalling Technology, 7076S).

### RNA extraction and real-time quantitative PCR

For cultured myoblasts and myotubes, total RNA was extracted using PureLink RNA Mini Kit (Ambion, 12183018A). For muscle tissues, total RNA was extracted using RNeasy Fibrous Tissue Mini Kit (Qiagen, MD, USA, 74704). cDNAs were synthesised using PrimeScript RT Reagent Kit (Invitrogen, TAKARA, RR037A) in a 20 μl reaction containing 0.2–0.5 μg of total RNA. Real-time quantitative PCR (RT-qPCR) was performed in a 15 μl reaction containing 0.5–1 μl of cDNA using iQSYBRGreen (Bio-Rad, CA, USA) in a Bio-Rad iCycler. PCR was performed by initial denaturation at 95°C for 5 min, followed by 40 cycles of 30 s at 95°C, 30 s at 60°C and 30 s at 72°C. Specificity was verified by melting curve analysis and agarose gel electrophoresis. The threshold cycle (C_t_) values of each sample were used in the post-PCR data analysis. Gene expression levels were normalised against the following housekeeping genes: β-tubulin for muscle tissues and RPLP0 for cultured myoblasts and myotubes. Real-time PCR primers are listed in electronic supplementary material (ESM) Table 1.

### Glucose uptake assay

The glucose uptake assay was performed on in vitro differentiated mouse and human myotubes in a 96-well plate using the Glucose Uptake Cell-Based Assay Kit (Cayman Chemical, MI, USA, catalogue no. 600470) according to the manufacturer’s instructions with minor modifications. On the day of the assay, culture media were replaced with 200 μl of glucose-free DMEM (Gibco, catalogue number 11966-025) and incubation was carried out for 2 h. Then, the medium was replaced with 100 μl of new glucose-free DMEM in the presence or absence of 100 nmol/l of insulin for 15–20 min. Subsequently, 100 μl of new glucose-free DMEM containing fluorescent 2-(*N*-[7-nitrobenz-2-oxa-1,3-diazol-4-yl]amino)-2-deoxyglucose at a final concentration of 150 μg/ml was added. Incubation was carried out in the dark for an additional 15 min in a tissue culture incubator. The medium was then removed and the myotubes were washed once with 200 μl of ice-cold PBS. After adding 100 μl of new ice-cold PBS to the myotubes, fluorescent intensity was immediately determined using the fluorescent plate reader (FilterMax F3&F5 Multi-Mode Microplate Reader; Molecular Devices, CA, USA). Results are presented with NT siRNA-transfected myotubes without insulin stimulation arbitrarily set as 1.

### Seahorse analysis

In vitro differentiated mouse and human myotubes in 24-well plates were used. Mitochondrial respiration analyses of myotubes were performed by the Islet, Oxygen consumption, Mass Isotopomer flux Core (IOMIC) at Yale using Seahorse XFPro (https://www.agilent.com/en/product/cell-analysis/real-time-cell-metabolic-analysis/xf-software/seahorse-wave-pro-software-2007523).

### Immunoprecipitation

To prepare antibodies, 50 μl (packed volume) of ChIP grade Dynabeads Protein G (Invitrogen, Thermo Scientific, 10004D) were washed twice with 1 ml of immunoprecipitation buffer (0.5% Triton X-100, 150 mmol/l NaCl, 10 mmol/l Tris HCl at pH 7.5, and 10 mmol/l EDTA), followed by incubation with 5 μg of rabbit polyclonal anti-TET3 (Active Motif, 61395), rabbit polyclonal anti-PGC-1α (Novus, NBP1-04676) or preimmune rabbit IgG in 300 μl of immunoprecipitation buffer at 4°C overnight. Antibody-bound beads were pelleted and kept on ice until use. To prepare lysate from muscle tissues, PBS-washed gastrocnemius muscle (GAS) tissues, freshly isolated from mice, were homogenised using a grinding tube in 1 ml of freshly prepared gentle lysis buffer (0.5% Triton X-100, 10 mmol/l NaCl, 10 mmol/l Tris HCl at pH 7.5, 10 mmol/l EDTA and 1× protease inhibitor cocktail). After centrifugation at 12,000 *g* at 4°C for 15 min to remove insoluble materials, 5 mol/l of NaCl was added to a final concentration of 200 mmol/l, and the lysate was transferred to a tube containing antibody/preimmune IgG-coated beads (250 μl of lysate per immunoprecipitation). Immunoprecipitation was carried out at 4°C for 4 h. Following this, beads were quickly washed twice with 1 ml of cold immunoprecipitation buffer and washed an additional three times by rotating at 4°C for 5 min each time. After the final wash, residual liquid was completely removed and the beads were eluted with 16 μl of 2 × SDS buffer (containing 1× phosphatase inhibitor cocktail and 1× protease inhibitor cocktail) at 100°C for 5 min. Eluant was loaded (10 μl per gel well) onto a 4–15% gradient SDS gel (Bio-Rad, 456-8086). For western blot analysis, anti-TET3 (Active Motif, 61395) and anti-PGC-1α (Novus, NBP1-04676) were used. The secondary antibodies used were Rabbit IgG TrueBlot (1:1000, Rockland, 18-8816-33). These unique HRP-conjugated monoclonal secondary antibodies enable detection of immunoblotted target proteins without hindrance by interfering immunoprecipitating immunoglobulin heavy and light chains.

### RNA-Seq and data analysis

Total RNAs were extracted from GAS tissues of 14-week-old RC-fed WT and mKD mice using RNeasy Fibrous Tissue Mini Kit (Qiagen, 74704). RNA-Seq library preparation and sequencing were conducted at Yale Stem Cell Center Genomics Core facility through poly A enrichment (lllumina TruSeq Stranded mRNA Library Prep Kit). Differential expression analysis between two different groups was performed using Partek Flow software, version 9.0.20.0622 (Partek, St Louis, MO, USA; https://www.partek.com/partek-flow/). Genes with a false discovery rate (FDR) below 0.05 and absolute fold change over 1.0 were analysed with Ingenuity Pathway Analysis using IPA software (Qiagen). Sequences are available from the Gene Expression Omnibus (https://www.ncbi.nlm.nih.gov/geo/) with accession number GSE224042.

### Statistical analysis

The number of independent experiments and the statistical analysis for each figure are indicated in the legends. All statistical analyses (except RNA-seq which was performed using DESeq2 software) were performed using GraphPad Prism version 8 for Windows (GraphPad Software, La Jolla California USA; www.graphpad.com) and are presented as mean ± SEM. Two-tailed Student’s* t* tests (or as otherwise indicated) were used to compare means between groups. *p*<0.05 was considered significant.

## Results

### TET3 expression is elevated in skeletal muscles of humans and mice with diabetes

**Fig. 1:**
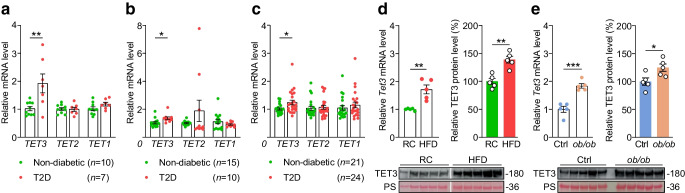
TET3 expression is increased in muscles of humans with diabetes and muscles of HFD-fed mice. (**a**) Relative *TET1*, *TET2* and *TET3* mRNA levels in muscle tissues from humans with diabetes (age 60.0±4.8 years, BMI 31.8±6.5 kg/m^2^) or without diabetes (age 59.6±5.0 years; BMI 27.4±5.4 kg/m^2^) (from the GSE22435 dataset). (**b**) Relative *TET1*, *TET2* and *TET3* mRNA levels in muscle tissues from humans with diabetes (age 51.5±3.6 years, BMI 30.8±2.5 kg/m^2^) or without diabetes (age 37.8±2.9 years, BMI 25.2±0.8 kg/m^2^) (from the GSE25462 dataset). (**c**) Relative *TET1*, *TET2* and *TET3* mRNA levels in myocytes from women with diabetes (age 46–63 years, BMI 24–33 kg/m^2^) or without diabetes (age 41–63 years, BMI 24–35 kg/m^2^) (from the GSE81965 dataset). (**d**) qPCR analysis and immunoblot of TET3 in GAS muscles from RC- and HFD-fed mice. The lane of each blot represents an individual mouse; molecular size in kDa is shown. (**e**) qPCR and immunoblot of TET3 in GAS muscles from control and *ob*/*ob* mice at the age of 10 weeks. All data are presented as mean ± SEM. *p<0.05, **p<0.01, ***p<0.001 (two-tailed Student’s *t* test). Ctrl, control; PS, Ponceau S; T2D, type 2 diabetes

We performed data mining on publicly available datasets (Gene Expression Omnibus). The expression of *TET3* (but not *TET1* and *TET2*) mRNA was significantly increased in skeletal muscle tissues of humans with diabetes as compared with non-diabetic control counterparts (Fig. 1a,b) [37, 38]. Increased *TET3* expression was also detected in myocytes from individuals with diabetes (Fig. 1c) [39]. Next, we asked whether muscle TET3 expression would change in mice with obesity and diabetes. Thus, we subjected mice to HFD feeding for 12 weeks, starting at the age of 6 weeks. Age-matched mice fed RC were used as a control. Compared with control mice, the HFD-fed mice developed obesity and impaired glucose metabolism, as judged by a significant increase in body weight and fat mass accompanied by a decrease in lean body mass and an increase in fasting glucose level (ESM Fig. 1). To assess TET3 expression, GAS muscle, composed of both slow-twitch type I oxidative and fast-twitch type II glycolytic fibres, was isolated, followed by qPCR and immunoblotting analyses. TET3 expression was significantly increased in muscle of HFD-fed mice as compared with RC-fed mice, at both the mRNA and protein level (Fig. 1d). The specificity of TET3 antibody has been previously validated [36, 40, 41]. Similar observations were made in *ob*/*ob* mice, a genetic mouse model of obesity and type 2 diabetes (Fig. 1e). The strong positive correlation between obesity/diabetes and increased muscle TET3 expression in both humans and mice suggests a role for TET3 in regulation of muscle insulin sensitivity.

### Muscle-specific TET3 knockdown enhances insulin sensitivity

To determine whether TET3 regulates muscle insulin sensitivity, we created mice with muscle-specific *Tet3* knockout (mKD) by breeding TET3 floxed mice (*Tet3*^fl/fl^) with transgenic mice (HSA-Cre79) expressing Cre recombinase under the control of HSA promoter, which has been shown to enable muscle-specific expression of transgenes [42, 43]. The WT TET3 floxed littermates were used as controls.

**Fig. 2:**
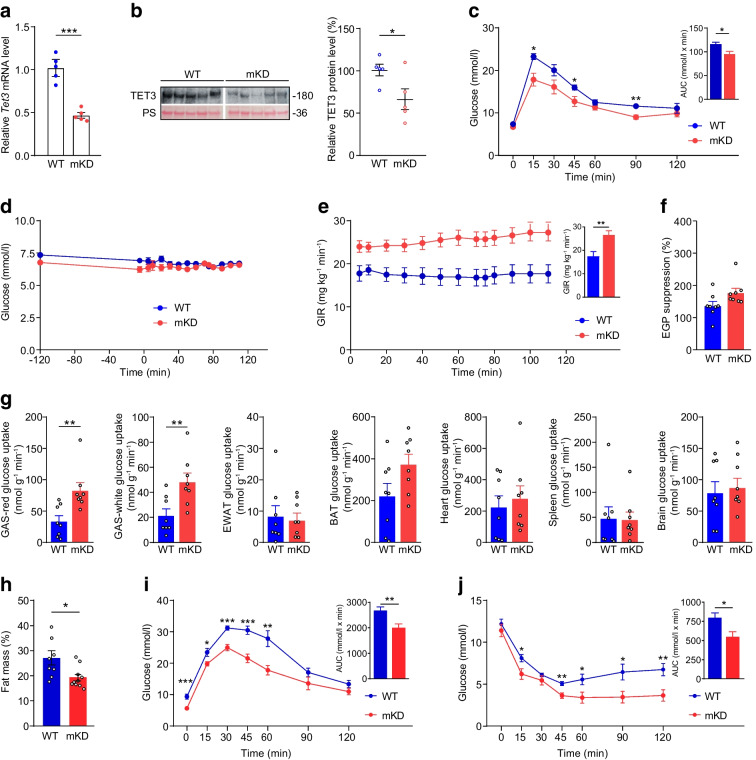
Muscle TET3 knockdown enhances insulin sensitivity. (**a**) qPCR of *Tet3* mRNA in GAS from WT and mKD mice at the age of 12 weeks. *n*=5 mice for each genotype. (**b**) Immunoblot of TET3 protein in GAS from WT and mKD mice at the age of 12 weeks. Each lane represents an individual mouse, with TET3 protein quantification shown. (**c**) GTT following 14 h overnight fasting of WT and mKD mice at the age of 10 weeks. *n*=8 mice in each group. (**d**–**g**) Hyperinsulinaemic–euglycaemic clamp studies from WT and mKD mice at the age of 12 weeks. *n*=8 animals in each group. (**h**) Per cent body fat of WT (*n*=8) and mKD (*n*=10) mice after exposure to HFD for 10 weeks. (**i**, **j**) Results of GTT (**i**) and ITT (**j**) of WT (*n*=6) and mKD (*n*=8) mice after exposure to HFD for 10 weeks. All data are presented as mean ± SEM. *p<0.05, **p<0.01, ***p<0.001 (**a**, **b**, **f**, **g** and **h**, two-tailed Student’s *t* test; **c**–**e** and **j**, two-way ANOVA with Sidak post-test). BAT, brown adipose tissue; EGP, endogenous glucose production; EWAT, epididymis white adipose tissue; GIR, glucose infusion rate; PS, Ponceau S

Muscle TET3 expression was reduced by ~50% at the mRNA level (Fig. 2a) and by ~25% at the protein level (Fig. 2b) in mKD as compared with WT littermates. The residual expression of TET3 in muscle extracts could be attributed to an incomplete deletion of the floxed allele in a fraction of myocytes and/or to the presence of other cell types in the tissue [44]. To exclude the potential for functional compensation of TET3 knockdown, we also examined the expression of other TET family isoforms. While TET1 expression in skeletal muscles was negligible, TET2 expression was abundant and was not altered by TET3 deletion (ESM Fig. 2). Total body weight, fat mass and lean body mass were similar when comparing mKD mice with WT littermates (ESM Fig. 3a,b) and indirect calorimetry experiments showed no differences in food intake, energy expenditure or locomotion (ESM Fig. 3c). However, when subjected to a GTT, the mKD mice showed an enhanced glucose tolerance as compared with WT littermates (Fig. 2c). To determine the cause of alterations in blood glucose more directly, hyperinsulinaemic–euglycaemic clamp studies were performed. Compared with WT littermates, the mKD mice showed significantly higher glucose infusion rate required to maintain euglycaemia (Fig. 2d,e), reflecting increased whole-body insulin sensitivity. This was not due to increased insulin-stimulated suppression of endogenous glucose production (Fig. 2f) but was due to increased insulin-stimulated glucose uptake into skeletal muscle (Fig. 2g). Collectively, these results demonstrate that muscle-specific TET3 knockdown increases muscle and whole-body insulin sensitivity.

### mKD mice are resistant to diet-induced insulin resistance

mKD and WT mice were subjected to HFD for 10 weeks, starting at the age of 6 weeks. The mKD mice gained less body fat than WT mice (Fig. 2h), with no difference in lean mass between the groups (data not shown). In addition, mKD mice were more glucose tolerant and had a better insulin sensitivity (Fig. 2i,j). These results further support the importance of muscle TET3 in modulation of whole-body glucose homeostasis.

### TET3 affects mitochondrial pathway gene expression

To gain a mechanistic insight into TET3-mediated regulation of muscle insulin sensitivity, we performed genome-wide expression profiling (RNA-seq) on RNA isolated from GAS muscles of mKD and WT littermates fed on RC. We performed Ingenuity Pathway Analysis on our RNA-seq data, using a cut-off of 1.2-fold change (*p*<0.05) in gene expression. We acknowledge that many common, complex disorders are characterised by modest but coordinated changes in expression of multiple genes of a biological pathway. Indeed, Gene Set Enrichment Analysis (GSEA) was previously used to successfully identify a subset of genes involved in oxidative phosphorylation whose expression was co-ordinately downregulated (20–50%) in muscles of humans with type 2 diabetes [18]. Importantly, this set of genes are also targets of PGC-1α, underscoring the long-standing notion of repression of the PGC-1α-dependent mitochondrial program in muscles of diabetic individuals [9, 16–19]. Our RNA-seq studies revealed profound gene expression changes induced by muscle *Tet3* knockout as compared with controls (ESM Fig. 4a and ESM Table 2). Pathway analysis identified ‘Mitochondrial Function’ and the ‘PPARα pathway’ to be among the top biological processes affected by TET3 (ESM Fig. 4b). By examining specifically how TET3 influenced the expression of genes in the mitochondrial pathway, we noticed a set of 31 mitochondrial respiration genes that were co-ordinately upregulated by at least 1.2-fold (*p*<0.05) in mKD vs WT mice (ESM Fig. 4c). As PGC-1α is a master regulator of mitochondrial biogenesis and function, we hypothesised that PGC-1α might be an important downstream target of TET3.

### TET3 post-transcriptionally affects PGC-1α expression

**Fig. 3:**
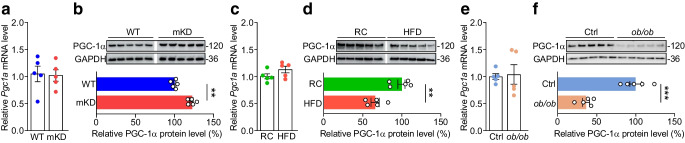
TET3 negatively regulates PGC-1α expression at the post-transcriptional level. (**a**) qPCR of *Pgc1a* mRNA in GAS tissues isolated from WT and mKD mice. (**b**) Immunoblot of PGC-1α in GAS tissues isolated from WT and mKD mice. (**c**) qPCR of *Pgc1a* in GAS tissues isolated from RC- and HFD-fed mice. (**d**) Immunoblot of PGC-1α in GAS tissues isolated from RC- and HFD-fed mice. (**e**) qPCR of *Pgc1a* in GAS tissues isolated from age-matched control and *ob*/*ob* mice. (**f**) Immunoblot of PGC-1α in GAS tissues isolated from age-matched control and *ob*/*ob* mice. Data are presented as mean ± SEM,* n*=5 mice in each group. **p<0.01, ***p<0.001 (two-tailed Student’s *t* test). Ctrl, control

As a crucial metabolic node, PGC-1α is subjected to both transcriptional and post-transcriptional regulation [45–47]. Our RNA-seq analysis did not detect a significant change in PGC-1α expression at the mRNA level in muscles of mKD vs WT mice (ESM Table 2). However, a change at the protein level could not be excluded. Thus, RNA and protein were isolated from muscles of mKD and WT mice and analysed. Consistent with our RNA-seq data, no significant change was detected in *Pgc1a* (also known as *Ppargc1a*) mRNA in muscles of mKD vs WT mice (Fig. 3a). However, there was a modest (~25%) but significant increase in PGC-1α protein in mKD mice (Fig. 3b). These results are in line with previous reports that physiologically relevant increases (~25%) in PGC-1α protein enhance muscle insulin sensitivity [9, 13–15], given that mKD mice also show increased insulin sensitivity (Fig. 2). Importantly, when muscles from mice were examined, we observed a ~25% and ~60% decrease in PGC-1α protein in HFD- vs RC-fed and *ob*/*ob* vs control mice, respectively, with no changes at the mRNA level (Fig. 3c–f). Collectively, our results suggested a post-transcriptional regulation of PGC-1α expression by TET3 in myocytes.

### TET3 affects mitochondrial respiration and insulin sensitivity in myocytes

**Fig. 4:**
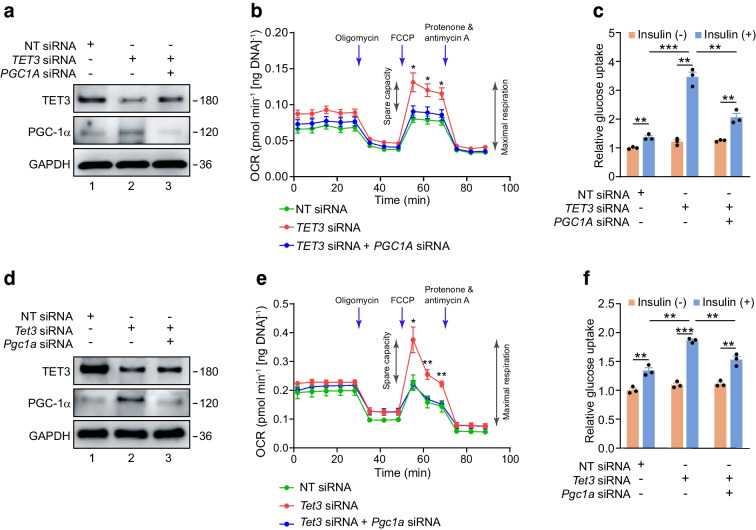
PGC-1α is required for TET3-mediated regulation of insulin sensitivity. (**a**) Immunoblot of TET3 and PGC-1α from human primary myotubes transfected with NT siRNA, *TET3* siRNA or *TET3* siRNA plus *PGC1A* siRNA. (**b**) Mitochondrial respiration of human primary myotubes treated as in (**a**). *n*=8 in each group. (**c**) Glucose uptake of human myotubes transfected with NT siRNA, *TET3* siRNA or *TET3* siRNA plus *PGC1A* siRNA as in (**a**) in the absence (−) or presence (+) of insulin at 100 nmol/l. Results are presented as relative glucose uptake with values in the absence of insulin set as 1. *n*=3 in each group. (**d**) Immunoblot of TET3 and PGC-1α from mouse C2C12 myotubes transfected with NT siRNA, *Tet3* siRNA or *Tet3* siRNA plus *Pgc1a* siRNA. (**e**) Mitochondrial respiration of mouse C2C12 myotubes treated as in (**d**). (**f**) Glucose uptake of mouse C2C12 myotubes transfected with NT siRNA, *Tet3* siRNA or *Tet3* siRNA plus *Pgc1a* siRNA as in (**d**) in the absence (−) or presence (+) of insulin at 100 nmol/l. *n*=3 in each group. Data are representative of two independent transfection experiments. *p<0.05, **p<0.01, ***p<0.001 (**b** and **e**, two-way ANOVA with Sidak post-test; **c** and **f**, two-tailed Student’s *t* test). FCCP, carbonyl cyanide-4 (trifluoromethoxy) phenylhydrazone; OCR, oxygen consumption rate

There is substantial evidence from human and animal studies that mitochondrial respiration is intrinsically coupled to muscle insulin sensitivity [48]. Notably, a ~25% increase of PGC-1α protein in muscle promotes insulin-sensitising effects including enhanced mitochondrial respiration and insulin-stimulated glucose uptake [9, 13–15, 18]. Our studies revealed that mKD mice exhibit increased insulin sensitivity (Fig. 2) with a concomitant increase in PGC-1α protein (Fig. 3b). Given the inverse relationship between TET3 and PGC-1α protein levels observed in human and mouse skeletal muscle tissues, we asked whether reducing TET3 protein levels in myocytes using siRNAs specifically targeting *TET3* mRNA for degradation would elevate PGC-1α protein level thereby enhancing mitochondrial respiration and insulin-stimulated glucose uptake. The siRNAs (*TET3* siRNA and *Tet3* siRNA for human and mouse genes, respectively) and the control non-targeting siRNA (NT siRNA) have been previously documented [40]. We also asked whether bringing PGC-1α protein levels back down to basal levels via siRNA-mediated knockdown in *TET3* siRNA-treated myocytes would abolish these effects. As expected, the PGC-1α protein level increased in human primary myotubes transfected with *TET3* siRNA as compared with NT siRNA (Fig. 4a). Co-transfection with *TET3* siRNA and *PGC1a* siRNA restored PGC-1α protein to basal levels (Fig. 4a). While reducing the TET3 protein level (which led to increased PGC-1α protein level, Fig. 4a) increased mitochondrial maximal respiration and spare respiration capacity, co-transfection with *TET3* siRNA and *PGC1a* siRNA (which restored PGC-1α protein to basal levels) abolished these effects (Fig. 4b). Likewise, insulin-stimulated glucose uptake increased by 1.4- and 3.5-fold in NT siRNA and *TET3* siRNA-transfected myotubes, respectively. This insulin-sensitising effect of TET3 knockdown was abrogated when PGC-1α protein was restored to basal levels (Fig. 4c). Similar results were obtained from mouse C2C12 myotubes (Fig. 4d–f). Based on these results we conclude that TET3 negatively regulates mitochondrial respiration and insulin sensitivity in myocytes and that PGC-1α is a major mediator of this regulation.

### TET3 destabilises PGC-1α protein

**Fig. 5:**
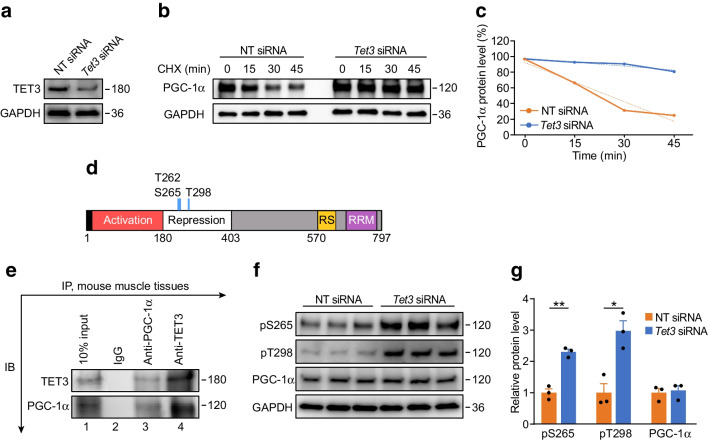
TET3 interacts with and destabilises PGC-1α. (**a**, **b**) C2C12 myoblasts were transfected with NT siRNA or *Tet3* siRNA. After 48 h, proteins were isolated and TET3 expression was measured by immunoblotting (**a**). To perform time course analysis, CHX was added at a final concentration of 50 μg/ml and proteins were harvested at the indicated time points, followed by immunoblotting for PGC-1α and GAPDH (**b**). (**c**) Quantification of (**b**); the dotted line indicates the trendline. (**d**) Schematic of PGC-1α protein domain organisation, showing the activation domain, repression domain, arginine–serine-rich domain (RS) and RNA recognition motif (RRM). Numbers represent amino acids. The blue vertical lines represent phosphorylation at Thr262, Ser265 and Thr298. Not drawn to scale. (**e**) Mouse GAS tissues were used for immunoprecipitation using preimmune IgG, anti-TET3 or anti-PGC-1α. Representative immunoblots are shown. (**f**) C2C12 myoblasts were transfected with NT siRNA or *Tet3* siRNA as in (**a**). After 48 h, proteins were isolated and analysed by immunoblotting using antibodies specific for total PGC-1α and PGC-1α phosphorylated at S265 and T298, respectively. (**g**) Quantification of (**f**). Data are presented as mean ± SEM. *p<0.05, **p<0.01 (two-tailed Student’s *t* test). Data are representative of two independent transfection experiments. IB, immunoblotting; IP, immunoprecipitation

Because PGC-1α regulates mitochondrial respiration and insulin sensitivity and because TET3 knockdown affected these processes without altering the mRNA abundance of PGC-1α, we hypothesised that TET3 affects PGC-1α stability. Thus, TET3 was knocked down in C2C12 myoblasts (Fig. 5a), followed by time course analysis of PGC-1α in the presence of CHX, a protein synthesis inhibitor. PGC-1α became more stable in *Tet3* siRNA-transfected as compared with NT siRNA-transfected cells (Fig. 5b). The levels of PGC-1α were reduced to ~80% and 20% of steady-state levels in *Tet3* siRNA-treated and NT siRNA-treated cells, respectively (Fig. 5c), suggesting that TET3 knockdown leads to stabilisation of PGC-1α.

PGC-1α is known to be targeted for ubiquitin-mediated proteolysis [49, 50]. Phosphorylation of PGC-1α at Thr262, Ser265 and Thr298 (Fig. 5d) by p38 mitogen-activated protein kinase (MAPK) results in an increase in its activity and protein stability [51–53]. We hypothesised that a complex between TET3 and PGC-1α might interfere with phosphorylation on these sites, accelerating PGC-1α degradation. First, we tested whether TET3 forms a complex with PGC-1α. We prepared lysates from mouse GAS muscle tissues, followed by immunoprecipitation using antibodies specific for TET3 [34, 54, 55] and PGC-1α, respectively. When TET3 was immunoprecipitated using anti-TET3 antibodies, we observed an enrichment of PGC-1α in the TET3-containing protein complex (Fig. 5e). Reciprocally, TET3 was readily detected in PGC-1α -containing protein complex (Fig. 5e). These results support complex formation between TET3 and PGC-1α in myocytes. Next, we analysed effects of TET3 knockdown on phosphorylation at S265 and T298 in C2C12 myoblasts using antibodies that specifically recognise these sites [53]. When TET3 was downregulated (Fig. 5a), increased phosphorylation at both sites (pS265 and pT298) was observed (Fig. 5f,g). Based on these results, we conclude that TET3 induces PGC-1α degradation at least in part by inhibiting phosphorylation at both sites.

## Discussion

In this work we show that TET3 plays an important role in the regulation of skeletal muscle insulin sensitivity. We identify PGC-1α as a major downstream effector of TET3 in the regulation of mitochondrial respiration and insulin-stimulated glucose uptake in both human and mouse myocytes. PGC-1α has previously been shown to be activated and stabilised by p38 MAPK phosphorylation at Thr262, Ser265 and Thr298 [51–53]. We demonstrate that TET3 complexes with PGC-1α and prevents its phosphorylation on Ser265 and Thr298, thereby accelerating protein degradation. This TET3-mediated post-translational regulation of PGC-1α in muscle insulin sensitivity is further supported by our observation that in skeletal muscles of humans with type 2 diabetes and mouse models of type 2 diabetes, there is an increase in TET3 expression and a decrease in PGC-1α protein as compared with non-diabetic controls.

Despite extensive studies of TETs in development, stem cells, malignancies and immunity [21, 56], their roles in metabolic regulation have just begun to be recognised. In mouse adipocytes, TET1 was found to act in concert with histone deacetylase 1 to epigenetically suppress thermogenic gene transcription. Phenotypically, adipocyte-specific *Tet1* knockout increased energy expenditure and protected against diet-induced obesity and insulin resistance [27]. Likewise, adipose-specific deletion of all three *Tet* genes in mice enhanced β-adrenergic responses, increased energy expenditure, and prevented obesity [28]. In pancreas beta cells, eliminating TET2 reduced pathological immune cell activation and beta cell killing during type 1 diabetes [57]. We reported a chronic increase in TET3 expression in the livers of humans and mice with type 2 diabetes and that TET3 induced *Hnf4a* promoter demethylation leading to heightened hepatic glucose production and hence hyperglycaemia [34, 35]. In addition, we documented that CRISPR-mediated genetic ablation of *Tet3* specifically in AgRP neurons in the mouse hypothalamus induced hyperphagia, systemic insulin resistance, obesity and type 2 diabetes [36]. Mechanistically, TET3 deficiency led to AgRP neuron activation and coordinated upregulation of *Agrp*, *Npy* and the vesicular γ-aminobutyric acid (GABA) transporter gene *Slc32a1*. Specifically, we demonstrated a dynamic association of TET3 with the *Agrp* promoter in response to leptin signalling, which induced 5hmC modification and association of a chromatin-modifying complex leading to transcription inhibition [36]. In the current manuscript we uncover a new role for TET3 in energy homeostasis in yet another major metabolic tissue, the skeletal muscle. However, the model of action of TET3 in this tissue is completely different from that previously defined in other tissues and with other TET family members. Instead of regulating target gene expression at the epigenetic level, TET3 post-translationally targets PGC-1α protein for degradation through inhibiting its phosphorylation. Our data support the notion that TET3 and PGC-1α are in a complex together but do not address whether they directly interact with each other. Nor it is clear how TET3 inhibits PGC-1α phosphorylation, given that TET3 is not a phosphatase. Does PGC-1α contain a binding motif that supports TET3 association? Does association with TET3 lead to altered subcellular localisation of PGC-1α such that it cannot be phosphorylated? A clear understanding of the mechanism of action of TET3 in regulation of PGC-1α stability warrants future in-depth investigation. An additional limitation of our study is that GAPDH is not an optimal loading control for skeletal muscle due to the plasticity in the expression of glycolytic pathway genes.

Increased expression of TETs in peripheral metabolic organs (i.e. liver, adipose tissue, pancreas and skeletal muscle) appears to have adverse effects on metabolic homeostasis [27, 28, 34, 35, 57]. Interestingly, this does not seem to be the case in the central nervous system [36], highlighting tissue/cell-specific functions of TETs. Finally, dysregulation of PGC-1α has been connected to many human diseases, such as Huntington’s disease, amyotrophic lateral sclerosis, heart failure, and Duchenne muscular dystrophy [58–63]. Our results demonstrating that TET3 regulates PGC-1α may have a broader impact on the prevention and treatment of other human diseases.

### Supplementary Information

Below is the link to the electronic supplementary material.

Supplementary file1 (PDF 1448 KB)

Supplementary file2 (XLS 2820 KB)

## Data Availability

Sequences are available from the Gene Expression Omnibus with accession number of GSE224042. All other study data are included in the article and/or ESM.

## Abbreviations

AgRP: Agouti-related protein
CHX: Cycloheximide
GAS: Gastrocnemius (muscle)
HFD: High-fat diet
HNF4α: Hepatocyte nuclear factor 4α
HRP: Horseradish peroxide
HSA: Human α skeletal muscle actin
HSkM: Human skeletal myoblasts
MAPK: Mitogen-activated protein kinase
mKD: Muscle-specific *Tet3* knockout
NT siRNA: Non-targeting control siRNA
PGC-1α: Peroxisome proliferator-activated receptor-γ coactivator α
RC: Regular chow
RT-qPCR: Real-time quantitative PCR
TET: Ten-eleven translocation
WT: Wild-type
