# Supplementary material for: Skeletal muscle TET3 promotes insulin resistance through destabilisation of PGC-1α
Source: Diabetologia. 2024 Jan 13;67(4):724–37. doi: 10.1007/s00125-023-06073-5 (PMC10904493; doi:10.1007/s00125-023-06073-5)
Supplement: Supplementary file 1 — Supplementary file1 (PDF 1448 KB) [file 125_2023_6073_MOESM1_ESM.pdf]

Electronic supplementary material (ESM)

ESM Table 1: Primers sequences used for RT-qPCR.

Gene	Forward Primer	Reverse Primer
Rplp0 (mouse)	5'-GCTCCAAGCAGATGCAGCA-3'	5'-CCGGATGTGAGGCAGCAG-3'
Tet3 (mouse)	5'-CTTCCTATGGCTGGGAGTGAG-3'	5'-CTGCCTTGAATCTCCATGGTAC-3'
Pgc1a (mouse)	5'-AACCACACCCACAGGATCAGA-3'	5'-TCTTCGCTTTATTGCTCCATGA-3'

ESM Table 2: No difference in muscle PGC-1α expression between mKD and WT mice detected by RNA-Seq analysis (separate excel file).

ESM Figures

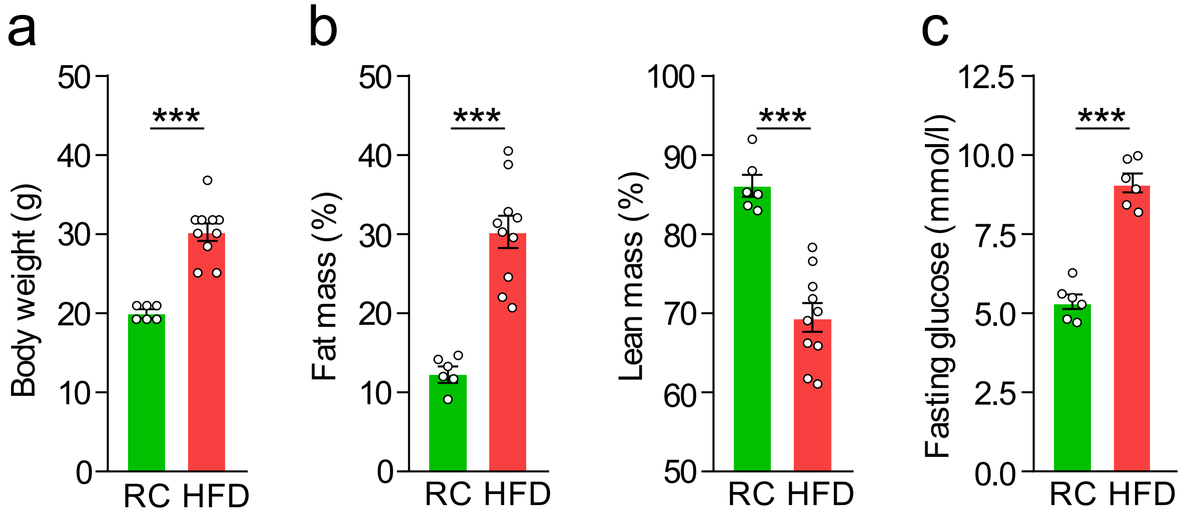

ESM Fig. 1: Body weight (**a**), body composition (**b**), and fasting glucose (**c**) of mice fed on HFD or RC. n=6-10 animals per group. Data are presented as mean ± SEM. ***p < 0.001. Two-tailed Student's t tests.

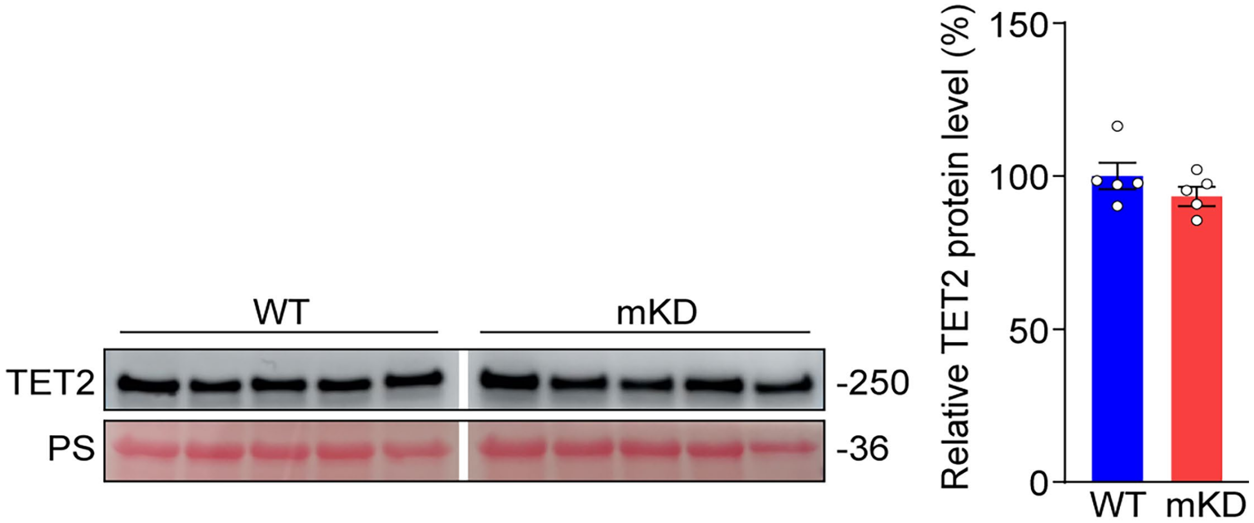

ESM Fig. 2: IB of TET2 protein in GAS from WT and mKD mice at the age of 12 weeks. Each lane represents an individual mouse, with TET2 protein quantification shown on the right. PS, Ponceau S. Data are presented as mean ± SEM. Two-tailed Student’s t tests.

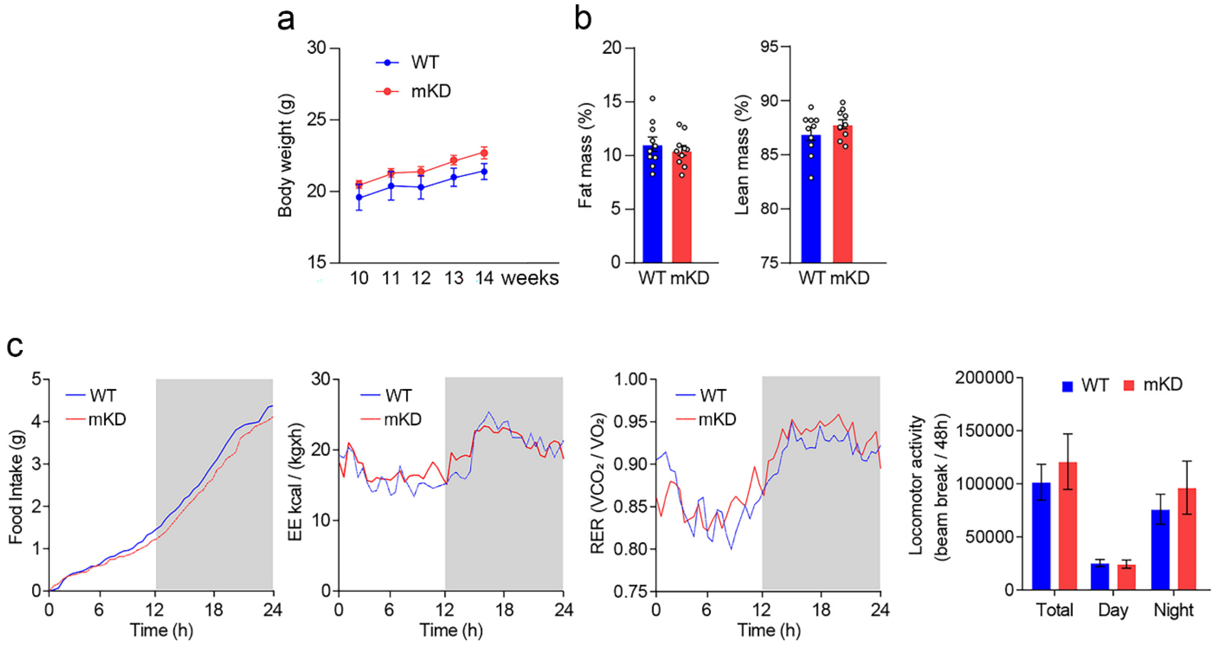

ESM Fig. 3: (**a**) Body weight of WT and mKD mice at the ages of 10-14 weeks. n=10 animals in each group. (**b**) Body composition by EcoMRI of WT and mKD mice at the age of 12 weeks. (**c**) Metabolic cage analyses at the age of 15-week old showing no difference in food intake, energy expenditure and locomotion between WT and mKD mice. n=8 animals per genotype. All data are presented as mean ± SEM. Two-tailed Student's t tests.

a

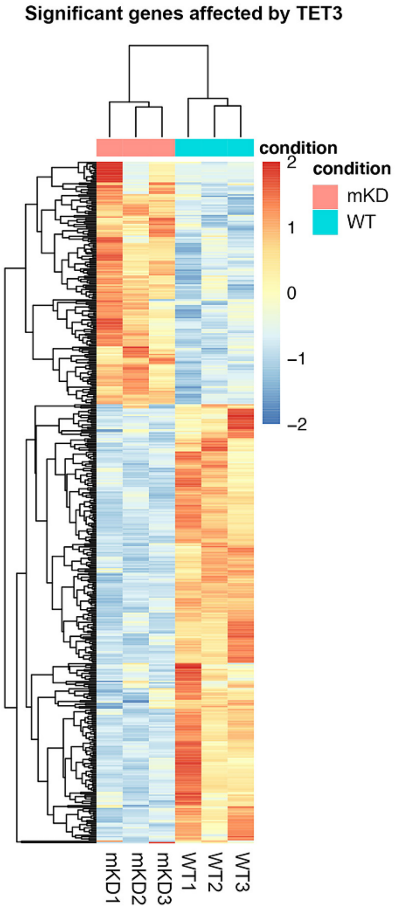

b

IPA analysis reveals biological processes affected by TET3

Name	p-value	Overlap
**Mitochondrial Function**	2.64E-08	18.3% 31/169
**Liver Proliferation**	3.31E-07	15.0 % 40/266
**Cardiac Fibrosis**	5.91E-07	14.5 % 41/282
**PPARa Pathway**	1.72E-06	21.1 % 20/95
**p53 Signalling**	3.38E-06	20.2 % 20/99

c

Mitochondrial respiration genes

Symbol	Entrez Gene Name	Log_2
Atp5e	ATP synthase, H+ transporting, mitochondrial F1 complex	0.498
ATP5F1D	ATP synthase F1 subunit delta	0.463
COX6A1	cytochrome c oxidase subunit 6A1	0.736
COX6A2	cytochrome c oxidase subunit 6A2	0.414
COX7A2	cytochrome c oxidase subunit 7A2	0.365
COX7A2L	cytochrome c oxidase subunit 7A2 like	0.395
COX7B	cytochrome c oxidase subunit 7B	0.315
COX8A	cytochrome c oxidase subunit 8A	0.636
Cox8b	cytochrome c oxidase subunit 8B	0.562
HSD17B10	hydroxysteroid 17-beta dehydrogenase 10	0.432
NDUFA2	NADH:ubiquinone oxidoreductase subunit A2	0.529
NDUFA3	NADH:ubiquinone oxidoreductase subunit A3	0.349
NDUFA6	NADH:ubiquinone oxidoreductase subunit A6	0.405
NDUFA7	NADH:ubiquinone oxidoreductase subunit A7	0.489
NDUFA11	NADH:ubiquinone oxidoreductase subunit A11	0.447
NDUFA12	NADH:ubiquinone oxidoreductase subunit A12	0.424
NDUFA13	NADH:ubiquinone oxidoreductase subunit A13	0.333
NDUFB2	NADH:ubiquinone oxidoreductase subunit B2	0.642
NDUFB4	NADH:ubiquinone oxidoreductase subunit B4	0.486
NDUFB7	NADH:ubiquinone oxidoreductase subunit B7	0.408
NDUFB8	NADH:ubiquinone oxidoreductase subunit B8	0.308
NDUFB11	NADH:ubiquinone oxidoreductase subunit B11	0.499
NDUFS7	NADH:ubiquinone oxidoreductase core subunit S7	0.512
NDUFV3	NADH:ubiquinone oxidoreductase subunit V3	0.38
PARK7	Parkinsonism associated deglycase	0.446
PRDX5	peroxiredoxin 5	0.359
PSENEN	presenilin enhancer, gamma-secretase subunit	0.441
SURF1	SURF1 cytochrome c oxidase assembly factor	0.439
TXN2	thioredoxin 2	0.343
UQCR11	ubiquinol-cytochrome c reductase, complex III subunit XI	0.816
UQCRQ	ubiquinol-cytochrome c reductase complex III subunit VII	0.449

ESM Fig. 4: TET3 knockdown alters mitochondrial pathway gene expression. (**a**) Heat map showing relative levels of genes in muscle of WT and mKD mice. Scale based on changes in log2 expression. n=3 biological replicates in each genotype. (**b**) Pathway analysis revealed “Mitochondrial Function” and “PPARa Pathway” being among the top biological processes affected by TET3. (**c**) Log2 expression levels of a set of 31 mitochondrial genes in muscle of mKD mice relative to WT.
